# International consensus criteria for diagnosing and staging hand–arm vibration syndrome

**DOI:** 10.1007/s00420-018-1359-7

**Published:** 2018-09-27

**Authors:** C. J. M. Poole, M. Bovenzi, T. Nilsson, I. J. Lawson, R. House, A. Thompson, S. Youakim

**Affiliations:** 1 Centre for Workplace Health, HSE’s Health and Safety Laboratory, Harpur Hill, Buxton, SK17 9JN UK; 2 Clinical Unit of Occupational Medicine, Department of Medical Sciences, University of Trieste, Trieste, Italy; 3 Occupational and Environmental Medicine, Department of Public Health and Clinical Medicine, Umea University, Umeå, Sweden; 4 Rolls-Royce, P O Box 31, Derby, DE24 8BJ UK; 5 Division of Occupational Medicine, Department of Medicine, St Michael’s Hospital and University of Toronto, Toronto, ON Canada; 6 Department of Medicine, University of British Columbia, Vancouver, BC Canada

**Keywords:** HAVS, Stockholm Workshop Scale, Delphi method, Health surveillance

## Abstract

**Purpose:**

In the 30 years since the Stockholm Workshop Scale (SWS) was published, the scientific literature on hand–arm vibration syndrome (HAVS) has grown and experience has been gained in its practical application. This research was undertaken to develop an up-to-date evidence-based classification for HAVS by seeking consensus between experts in the field.

**Methods:**

Seven occupational physicians who are clinically active and have had work published on HAVS in the last 10 years were asked to independently take part in a three-round iterative Delphi process. Consensus was taken when 5/7 (72%) agreed with a particular statement. Experts were asked to provide evidence from the literature or data from their own research to support their views.

**Results:**

Consensus was achieved for most of the questions that were used to develop an updated staging system for HAVS. The vascular and neurological components from the SWS are retained, but ambiguous descriptors and tests without adequately developed methodology such as tactile discrimination, or discriminating power such as grip strength, are not included in the new staging system. A blanching score taken from photographs of the hands during vasospastic episodes is recommended in place of self-recall and frequency of attacks to stage vascular HAVS. Methods with the best evidence base are described for assessing sensory perception and dexterity.

**Conclusions:**

A new classification has been developed with three stages for the clinical classification of vascular and neurological HAVS based on international consensus. We recommend it replaces the SWS for clinical and research purposes.

## Introduction

It has been 30 years since the Stockholm Workshop Scale (SWS) was published for the classification of hand–arm vibration syndrome (HAVS) (Gemne et al. 1987; Brammer et al. 1987). Although an improvement on the previous Taylor–Pelmear scale, it contains subjective terms such as ‘occasional’ and ‘frequent’ which can cause difficulty with vascular staging. Concern has previously been expressed about a scale that combines frequency of attacks with extent of blanching (Palmer and Coggon 1997). Factors such as the ambient temperature, whether protective clothing was being worn, or the worker’s ability to accurately report their symptoms can also cause difficulty with staging.

Since the SWS was published, various clinical and laboratory tests of vascular and sensorineural function have been studied, but their place in clinical practice is unclear. Stage 2 of the sensorineural component requires sensory perception to be reduced, but the modalities for testing and how loss should be determined are left to the assessing physician. Stage 3 refers to tactile discrimination, which could be interpreted as spatial orientation in the palm or two-point discrimination (2-PD) at the fingertips, but there are no standardised methods of doing this or normative data for comparison.

A modification to the SWS has been used in the UK which divides stage 2 into early and late (Lawson and McGeoch 2003; McGeoch et al. 2004; HSE 2005) but it lacks a scientific evidence base and assumes that neuropathology progresses in a linear, ordinal way. To assist with staging, subjective terms in the SWS such as occasional, frequent, intermittent and persistent have been defined; however, they lack an objective basis.

This study was undertaken to gather international consensus by a group of experts on how the SWS could be improved in the light of research that has been published since 1987 with the aim of developing a more evidence-based clinical staging system for HAVS. By so doing, the accuracy of diagnosis and the management of workers with HAVS should be improved. The Delphi method was selected to do this as it allows experts who are remote from one another to achieve consensus in a structured way about a problem in their own time and without the dominance of any one idea or personality.

## Methods

Nine clinically active experts who have had papers published on HAVS in peer-reviewed journals or written chapters on HAVS in textbooks in the last 10 years were invited to take part in a Delphi process to update the classification and staging system for HAVS. The project was led by CJMP who acted as the facilitator and set the questions, but he took no part in the voting. The experts, who were unknown to one another, were asked to independently answer specific questions about the SWS, HAVS and the management of workers with HAVS. Participants were sent pertinent references with their main findings with each question. They were asked to reference other relevant research to include data to support their responses and to show their reasoning by free text comments. They could also raise new questions.

Consensus was set at 5/7 (72%) experts in agreement. After each round, the experts were given the group’s results and given the opportunity to change their views in the light of the comments made by others or evidence from shared references or data that were new to them. When consensus could not be reached the question was reformulated to try to obtain an agreed view. If an agreed view could not be arrived at, the question was abandoned.

One of the questions that came out of the Delphi was the degree of association between the frequency and extent of blanching, so data previously described (Poole et al. 2016) on patients with HAVS referred to the Health and Safety Laboratory in England for high-level health surveillance with standardised quantitative sensory tests (QSTs) were re-analysed by case for frequency of vasospastic episodes per week and extent of blanching as described by Griffin. Results were presented as scatter plots and Spearman’s rho tests calculated for the dominant and non-dominant hands.

Another question was the number of sensory modalities that needed to be tested and whether it was necessary to include both thermal and vibration perception tests. To inform this decision, data from a previous study (Poole et al. 2016) were re-analysed to ascertain the proportions of cases with abnormalities of thermal (hot and cold) and vibration (31.5 and 125 Hz) perception. The method of scoring the results of the QSTs was the same as that reported by others (Lawson and McGeoch 2003; McGeoch et al. 2004, HSE 2005); however, non-specific scores of < 4 in each hand were ignored.

As the Delphi progressed it became apparent that we were likely to recommend the use of Semmes–Weinstein monofilaments and the Purdue pegboard. This being the case we needed to know the cut-off from normal of sensory perception using monofilaments in the fingers of asymptomatic non-vibration exposed male heavy manual workers and whether previously published normal data for the Purdue pegboard applied to heavy manual workers. Both questions were addressed by an expert re-analysing his previously published data on non-vibration-exposed maintenance workers as medians and interquartile ranges (Bovenzi et al. 2015). This was done as normal limit values by age and hand.

## Results

Seven out of the nine experts approached agreed to take part in the Delphi process. One of those who declined was no longer active in the field and the other could not be contacted. The experts were from four countries on three continents. All seven took part in all rounds of the Delphi. A fourth round was required to achieve consensus on the staging criteria for stage 2 neurological. The detailed comments made by each expert are not recorded here but were used to gain consensus, frame questions, to draw conclusions and to formulate the recommendations in this paper. Some of the references that were used to form an opinion and the supporting information contained within them are recorded in Tables 1 and 2.

**Table 1:** Questions for which consensus was obtained

Question	Round	Agree	Disagree	No opinion	References	Supporting information
The SWS for HAVS should be revised	1	7	0	0	Lawson (2016)	Arguments for and against in editorial and letter pages
Staging should continue to be divided into vascular and sensorineural components	1	7	0	0	Brammer et al. (1987); Stromberg et al. (1999); Bovenzi et al. (2011); Bovenzi et al. (2015); Nilsson et al. (2017)	Vascular and sensorineural effects may develop separately; cases of HAVS divide symptomatically into those with vascular, sensorineural or combined symptoms; two longitudinal studies have shown an exposure–response relationship for neurosensory effects and work ability; evidence from a systematic review that neurosensory effects begin about 5 years before vascular ones
Vascular staging should be based on the extent of blanching in preference to the frequency of blanching	1	6	1	0	Gemne et al. (1987); Palmer and Coggon (1997)	A scoring system is needed to improve the discriminatory power of staging by the SWS; no strong relation between frequency and extent of blanching; extent of blanching with a frequency subscript was proposed by one expert; analysis as part of the Delphi showed a low to moderate correlation between the frequency and extent of blanching
Photographs taken during an attack of blanching should be used for diagnostic vascular staging	1	7	0	0	Youakim (2008)	Author was the first person to recommend the use of photographs during a vasospastic episode to confirm the presence of blanching in keeping with Raynaud’s phenomenon in vibration-exposed workers
Subjective terms such as rarely, occasional, frequent, intermittent and persistent should where possible be replaced by more objective descriptors	1	7	0	0	Gemne et al. (1987); Brammer et al. (1987); Lawson and McGeoch (2003)	Frequency terms not defined in SWS; subjectively defined by Lawson and McGeoch 2003 and adopted by HSE 2005 with no adjustment for ambient temperature or whether gloves were worn
The method(s) and criteria used to determine loss of sensory perception should be defined	1	7	0	0	Brammer et al. (1987); Cederlund et al. (2003)	Tests used should be standardised, reliable and validated with normative data
The method(s) and criteria used to determine reduced manipulative dexterity should be defined	1	7	0	0	Cederlund et al. (2003); Rui et al. (2008)	Reduced dexterity with increasing exposure and with higher vascular and sensorineural stages; therefore, reasonable to include in the assessment. Test used should be standardised, reliable and validated with normative data
Because of its rarity and strong association with rheumatological disease trophic changes in the fingertips should be dropped from stage 4 of the SWS	1	5	2	0		No scientific evidence
There should be specialised centres where patients can be referred for the diagnosis and staging of HAVS	1	6	1	0	Lawson (2016)	Access to laboratory tests, nerve conduction tests, Doppler ultrasound or angiography is required for accurate diagnosis and staging of HAVS
When available, the results of angiography of the hand, forearm or arm should be taken into consideration for vascular staging	1	0	7	0	Thompson and House (2006); Poole and Cleveland (2016)	Occlusions of ulnar, radial, palmar and digital arteries have been identified but whether they were congenital or acquired was not known. Angiography should not be routinely undertaken in vascular HAVS
Reduced tactile discrimination, sometimes interpreted as spacial orientation of an object in the hand or two-point discrimination at the fingertips, should be dropped from the scale	2	6	1	0	Brammer et al. (1987); Bovenzi and Zadini (1989); Stromberg et al. (1998); Lundborg and Rosen (2004); Mason and Poole (2004); Rolke et al. (2013)	Methodology for testing 2-PD not standardised; no normal range for non-vibration exposed heavy manual workers; in one study gap detection was reduced in a group of workers with HAVS compared with controls and difference between groups increased as gap width increased; no evidence that 2-PD can discriminate between stages; 2-PD can distinguish between groups but not individuals
When available, the results of multi-segmental nerve conduction tests (that is not just across the wrist) should be taken into consideration for sensorineural staging	2	6	1	0	Bovenzi et al. (2000); Hirata and Sakakibara (2007); Lander et al. (2007)	Evidence from two cross-sectional studies and a specialist assessment centre of multi-focal neuropathy in the hand, median, ulnar and posterior interosseous nerves proximal to the hand in workers with HAVS
Stage 2 can be reliably separated into early and late on the basis of the frequency of blanching or the duration of sensorineural symptoms	2	0	7	0	Lawson and McGeoch (2003); McGeoch et al. (2004); HSE (2005)	No scientific evidence to support
Stage 2 can be reliably separated into early and late on the basis of the results of cold provocation tests or quantitative sensory tests	2	1	6	0	Lawson and McGeoch (2003); McGeoch et al. (2004; HSE (2005)	No scientific evidence to support
The cut-off between normal and abnormal dexterity should be taken from Agnew et al. (1988) as 2 SD below the mean and stratified by age	3	6	1	0	Agnew et al. (1988); Rui et al. (2008)	Data from 212 normal subjects age 40–85; reproduced in Lafayette Instrument user manual; dexterity inversely related to age; women more dextrous than men; there is a learning effect with repeated testing; Table 4
Because of its low sensitivity for the diagnosis or staging of HAVS and its predominant contribution coming from muscles in the forearm and the influence of confounding factors such as age and general health, grip strength should be dropped from the scale	3	6	1	0	Mahbub et al. (2015)	No evidence that weakness of grip or pinch force is specific to HAVS or correlates with stage
The new staging scale (Table 5) is more objective than the SWS	3	5	2	0		Delphi
Health surveillance for HAVS should be used for education, the evaluation of the effectiveness of controls and to prevent workers getting to stage 3 vascular or to stage 3 sensorineural of the new scale	3	7	0	0	HSE (2005)	Regulatory guidance
Judgements about fitness and safety to work should depend on the nature of the work, frequency and duration of the blanching and the ambient temperature in which the work is undertaken rather than reaching a particular stage on the scale	3	5	2	0	HSE (2005); Cooke and Kloss (2017)	Regulatory guidance and expert opinion; fitness and safety to work with vibrating tools should depend on the worker’s assessed ability to do their job safely, rather than when a specific stage of HAVS has been reached; workers have the right of self-determination provided they understand the risks they are incurring by continued exposure to HTV and the safety of others is not compromised
Stage 2 SN should be defined as sensory perception loss in two or more fingers as evidenced by two or more validated methods to include monofilaments, thermal aesthesiometry and vibrotactile thresholds	4	6	1	0	Brammer et al. (1987); Stromberg et al. (1998); Lindsell and Griffin (2003); Seah and Griffin (2008); House et al. (2009); Kurozawa et al. (2010); Bovenzi et al. (2011); Poole et al. (2016)	Traditional neurological methods have limited ability to separate cases by stage; several cross-sectional studies have shown abnormalities of thermal and vibration perception in the fingers of workers with HAVS compared with controls; QSTs more sensitive for detecting sensorineural abnormalities than conventional nerve conduction tests; there are standardised methods and normal ranges of thermal and vibration perception by age and sex; thermal and vibration perception may be affected differentially (see results); testing of one finger innervated by the median nerve and one by the ulnar nerve recommended as a minimum; evidence from one study that thermal and vibration QSTs can distinguish SN stage 1 from 2. Delphi; abnormalities in two modalities are more diagnostically secure than in one; abnormality in only one finger is more likely to be due to neuropathy proximal to the hand unless excluded by nerve conduction tests

**Table 2:** Questions for which consensus could not be obtained

Question	Round	Agree	Disagree	No opinion	References	Supporting information
In a case of vascular HAVS, an abnormal Allen’s test should be investigated by either Doppler or MR angiography according to local expertise and the availability of tests	2	4	3	0	Cooke (2003); Thompson and House (2006); Poole and Cleveland (2016)	Several case reports
When using the Purdue pegboard, it is sufficient for the worker to insert pegs into a board for each hand and both hands and not to do a more complex assembly with collars and washers	2	3	0	4		No scientific evidence
Pending further information, the cut-off between normal and abnormal sensory perception of the digit pulp by monofilaments should be taken as 1.4 g-f for male heavy manual workers	3	3	2	2	Schulz et al. (1998); Birke et al. (2000)	80% of subjects < 0.4 g-f for men > 55 years of age but only two of the 120 subjects were heavy manual workers; 1.4 g-f distinguished between normals and those with leprosy; perception threshold increased with age and heaviness of work; Table 3
An individual with primary Raynaud’s phenomenon which is detected at the pre-placement stage should not be exposed to hand-transmitted vibration	3	4	3	0	HSE (2005)	No scientific evidence; regulatory guidance

In a series of 100 vascular HAVS cases referred to a specialist centre for QSTs (Poole et al. 2016) scatter plots of frequency vs. extent of blanching showed no particular pattern, apart from groupings at a frequency of seven per week and a blanching score of 12. There were 10 cases with a blanching score of > 18 combined with a frequency of blanching > 3 per week. Correlation coefficients of 0.42 and 0.31 were obtained for frequency and extent of blanching for the dominant and non-dominant hands, respectively.

In a series of 161 patients with HAVS and abnormal QSTs (Poole et al. 2016) there were abnormalities of both thermal and vibration perceptions in 86 (53%); abnormalities of only thermal perception in 42 (26%) and abnormalities of only vibration perception in 33 (21%).

The results of sensory perception and dexterity testing of asymptomatic non-vibration-exposed heavy manual (maintenance) workers are shown in Tables 3 and 4. The Semmes Weinstein data were not normally distributed so medians and 95th percentiles are shown. The Purdue pegboard data were normally distributed so means and − 2SDs are shown.

**Table 3:** Normal limit values for sensory perception thresholds by Semmes Weinstein monofilaments in heavy manual workers not exposed to vibration

Age range in years (*n*)	Right index finger (g-f)	Right little finger (g-f)	Left index finger (g-f)	Left little finger (g-f)
25–35 (37)	Range 0.07–2.04 Median 0.07 95th centile 2.04	Range 0.07–2.04 Median 0.07 95th centile 0.4	Range 0.07–2.04 Median 0.07 95th centile 2.04	Range 0.07–2.04 Median 0.07 95th centile 2.04
36–45 (45)	Range 0.07–2.04 Median 0.07 95th centile 2.04	Range 0.07–2.04 Median 0.07 95th centile 2.04	Range 0.07–2.04 Median 0.07 95th centile 0.4	Range 0.07–2.04 Median 0.07 95th centile 2.04
46–66 (36)	Range 0.07–2.04 Median 0.4 95th centile 2.04	Range 0.07–2.04 Median 0.4 95th centile 2.04	Range 0.07–2.04 Median 0.4 95th centile 2.04	Range 0.07–2.04 Median 0.4 95th centile 2.04
All (118)	Range 0.07–2.04 Median 0.07 95th centile 2.04	Range 0.07–2.04 Median 0.07 95th centile 2.04	Range 0.07–2.04 Median 0.07 95th centile 2.04	Range 0.07–2.04 Median 0.07 95th centile 2.04

## Discussion

Consensus was achieved by clinical experts using the Delphi method on several issues related to the assessment and staging of HAVS, enabling a more evidence-based and objective classification to be developed in workers exposed to sufficient hand-transmitted vibration (HTV) to cause HAVS (Table 5). Although the correlation between frequency and extent of blanching was not high, it is recommended that a blanching score, as described by Griffin (1990) is used to stage vascular HAVS. This is an objective measure of the extent of vasospasm and should be taken from photographs of the hands in ventral and dorsal views during an attack of blanching with the arms elevated alongside the face. A colleague or friend of the worker would need to take the photographs. If the most severe attack has not been captured, then the scoring could be provisional pending additional photographs.

The photographs serve the purpose of confirming symptom description and that the blanching is of the type associated with Raynaud’s phenomenon (RP). Two colours such as blanching and cyanosis or blanching and hyperaemia make the diagnosis of RP more secure than just blanching alone (Maverakis et al. 2014). Therefore, in cases where only one symptom is present, or photographs are not available for review, the diagnosis could be qualified as ‘probable’. As the thumb is rarely blanched in HAVS this digit need not be considered, so the maximum score for each hand would be 24. A half score could be allocated to a phalanx blanched > 50% but less than 100%.

Scatter plots did not indicate any obvious distinction between frequent and non-frequent blanching; however, vasospasm occurring more frequently than once a day, or blanching lasting more than an hour in duration are of concern regardless of the stage and suggest the need to check the blood supply to the hands. A frequency subscript to the blanching score was considered but rejected, as whatever frequency was chosen to be frequent would be subjective and strongly influenced by the ambient temperature and clothing.

Cold intolerance, which may be a symptom of constitutional or acquired cold hypersensitivity, RP or arterial occlusion in the hands has not been included in the scale, but it is an important symptom for diagnosis and safety to work. The new scale does not distinguish between vascular HAVS and primary RP which remains the main differential diagnosis. The current vascular provocation tests will not distinguish between them, so their use for diagnostic purposes has ceased in some HAVS centres. There is evidence that finger systolic blood pressure after local cooling according to ISO 14835-2:2005 can reflect the severity of vascular HAVS with stronger reactions of the digital arteries in the fingers of those with greater blanching scores. Vascular provocation tests are used in some countries for compensation purposes to confirm an abnormal vascular response to cold in those with or without blanching.

Tactile discrimination has been dropped from the neurological component because there is no standardised technique for doing this and no comparative normative data. Grip strength is also not included because of its lack of discriminant power for neurological HAVS, although it remains an important measure when assessing safety to work. Digital nerve conduction is unreliable distal to the proximal phalanx due to the low amplitude of the sensory action potentials and in any case, there are no normal values for the digits. The consensus view of the best available evidence for how sensory perception should be assessed in the digits was to use two or more standardised methods such as Semmes–Weinstein monofilaments for the perception of touch, thermal aesthesiometry for hot and cold sensibility, and vibration perception at a minimum of two frequencies (31.5 Hz and 125 Hz, which are thought to stimulate two different populations of mechanoreceptors). In time, other methods with good evidence bases might become available to determine sensory loss.

There was uncertainty between the experts as to whether abnormality of sensory perception in only one finger was enough to diagnose sensory neuropathy due to HTV. On balance, it was thought that because HTV acts diffusely, and to avoid misdiagnosing a neuropathy proximal to the hand as HAVS, it was preferable to set the threshold as abnormality in at least one finger supplied by each of the median and ulnar nerves. The practical effect of requiring abnormalities of sensory perception by two methods and in two fingers will be to move some cases currently staged by the SWS as 2 SN to 1 SN.

The methods of testing should be standardised. There is an international standard for vibration perception testing (ISO 13091-2:2003) but not for thermal perception or monofilament testing. However, thermal perception testing has been well described (Lindsell and Griffin 2003) with evidence for keeping digital skin temperature above 22 °C and preferably above 26 °C, but it is acknowledged that by so doing this may not be reproducing the worker’s normal pathophysiological state at work. There is evidence that for thermal and vibration QSTs there is no need to control for age in the 20–65-year age range (Lindsell and Griffin 2003; Seah and Griffin 2008), or for skin thickness (Lundstrom et al. 2018). Ideally, multi-segmental nerve conduction tests of sensory and motor nerve conduction velocities, latencies and wave amplitudes should be undertaken to exclude large fibre neuropathy in the hand, wrist and forearm which may adversely affect the QSTs in the digits. The cause of any large fibre neuropathy could be due to vibration, trauma to the limbs, abnormal ergonomics whilst working, nerve entrapment, diabetes or other general medical problem.

Thresholds for the neurological component of HAVS should be based on normative data from appropriate controls. Abnormality should be set as 2 standard deviations (SD) from the mean or outside 5th or 95th percentiles of the controls, according to the distribution of the data. More work is required to determine the cut-off between normal and abnormal for monofilament perception in older heavy manual workers not exposed to vibration, but it is thought to lie between 1.4 and 2.0 g-f (Birke et al. 2000; Table 3). This is because the sensory perception threshold for monofilaments appears to rise (ie sensation reduces) with age and thickness or hardness of the epidermis, the latter being a consequence of heavy manual work unless gloves are regularly worn. It is recommended that all digits are tested with monofilaments on the pulps away from callosities and that the sensory threshold is taken as the lightest monofilament detected by the ‘2 out of 3’ method. The bend forces of the monofilaments should be validated at regular intervals.

Thermal perception can be measured for hot, cold or both modalities. Abnormality can be measured for each modality or as a thermal neutral zone. The merit of each method is uncertain. Vibration perception can be measured at two or more frequencies to include 31.5 Hz and 125 Hz, but the merit of measuring at more frequencies is unknown. The greatest increase in vibration perception threshold in workers with HAVS has been shown to be at 100 Hz (Rolke et al. 2013). Normative values for thermal and vibration perception have been published for manual workers (Lindsell and Griffin 2003, ISO 13091-2:2003; Seah and Griffin 2008) and unlike other methods have been shown to distinguish between stages 1 and 2 of sensorineural HAVS (Poole et al. 2016). Ideally, all fingers should be tested, but for reasons of efficiency, a compromise may need to be found between the time taken to do the tests and the additional information gained from them. The current perception threshold method is a QST that has been used mainly in Canada and Japan, but it has been shown to lack the discriminating ability to distinguish between SWS sensorineural stages 1 and 2 (House et al. 2009; Kurozawa et al. 2010), so this limitation needs to be borne in mind if using this method.

Provided proximal neuropathy has been excluded, ideally with the aid of nerve conduction tests, then QSTs should be an accurate method of determining sensory neuropathy for thermal and vibration perception, rather than as an expensive optional ‘add-on’ to clinical testing. The traditional clinical methods of testing for sensory perception with cotton wool, pinprick, vibrating tuning forks and 2-PD are unlikely to be sufficiently reliable on their own for the accurate diagnosis of stage 2N HAVS. Instead, we recommend screening with Semmes–Weinstein monofilaments and referral to a specialised centre of any case with a significant deterioration in bend force thresholds. If sensory perception is found to be abnormal then the Purdue pegboard should be used to test dexterity.

The Purdue pegboard test is a validated method for determining dexterity with good reliability and for which normal distributions have been published by age and sex (Agnew et al. 1988) and for male heavy manual workers (Table 4). For workers over the age of 50, a minimum of 10 pegs inserted with the dominant hand in 30 s; 10 with the non-dominant hand and 8 with both hands appear to be reasonable cut-offs from normal. Although not tested in a vocational setting, the additional assembly of collars and washers with the pegs is probably not necessary to determine whether dexterity is impaired in heavy manual workers. Causes of a loss of dexterity other than from sensorineural HAVS such as a tremor, pyramidal weakness, impaired visual acuity, cognitive deficit or intentionally slow movements are relevant to assessment, so careful observation of how the worker performs the test is as important as the absolute score. An abnormal Purdue pegboard score in the context of normal sensory perception suggests the finding is not related to neurological HAVS and, therefore, should not be taken into consideration when rating this component of the scale (Table 5).

**Table 4:** Normal limit values for dexterity test scores by Purdue pegboard in heavy manual workers not exposed to vibration

Age range in years (*n*)	Dominant hand	Non-dominant hand	Both hands
25–35 (37)	Range 13–19 Mean 15.3 − 2SD 12.4	Range 12–19 Mean 15.0 − 2SD 11.8	Range 11–15 Mean 13.1 − 2SD 10.5
36–45 (45)	Range 13–18 Mean 15.1 − 2SD 12.6	Range 12–17 Mean 14.7 − 2SD 12.6	Range 9–16 Mean 12.9 − 2SD 10.2
46–66 (36)	Range 10–18 Mean 14.3 − 2SD 10.3	Range 11–18 Mean 14.1 − 2SD 10.6	Range 8–15 Mean 12.3 − 2SD 9.0
All (118)	Range 10–19 Mean 14.9 − 2SD 11.7	Range 11–19 Mean 14.6 − 2SD 11.6	Range 8–16 Mean 12.8 − 2SD 9.9

**Table 5:**
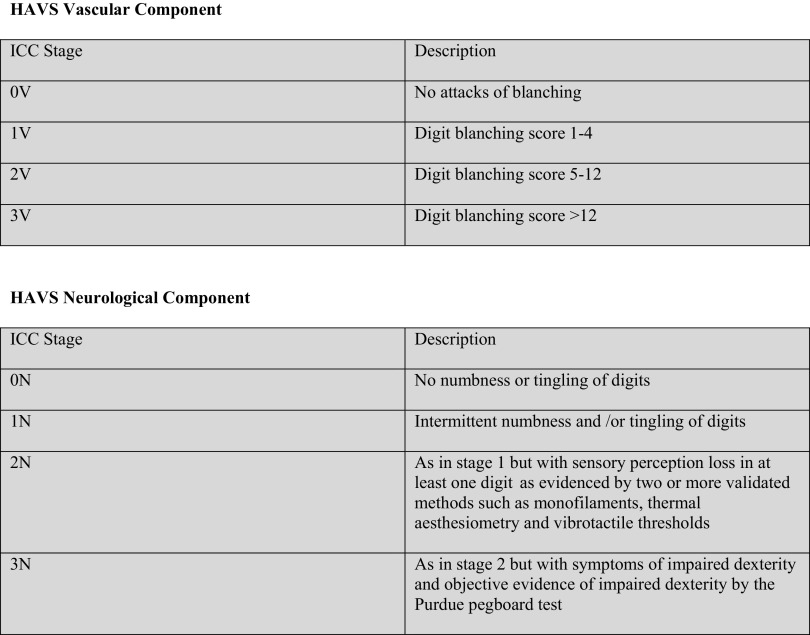
International Consensus Criteria (ICC) for the staging of HAVS

Each hand to be staged separately; Raynaud type blanching to be confirmed by a validated photograph with blanched skin clearly demarcated from unaffected skin; blanching score to be calculated from the photographs as 3 for proximal, 2 for middle and 1 for distal phalanx of each affected finger; neurosensory symptoms to be considered pathological if lasting > 20 min (Burstrom et al. 2009); sensory perception to be assessed on the pulps of two or more digits supplied by the median and ulnar nerves; standardised methods of testing must be followed for all tests and compared with appropriate controls

The strength of this research is that the views of international experts on HAVS and its staging have been captured independently. It does not necessarily reflect the views of any one expert. Where possible views were supported or influenced by evidence from the published literature or by the analysis of existing laboratory data in the UK and Italy. We are aware that QSTs, expertise in their interpretation and multi-segmental nerve conduction testing are not widely available in the community, but our recommendations have been guided by the scientific evidence rather than by practical considerations. For the first time, the criteria selected for staging are suitable for audit. We recommend that specialist centres are created with access to the full range of tests required for the diagnosis and staging of HAVS.

A limitation of the research is that the questions used for each round were formulated by the facilitator and some aspects of classification may not have been sufficiently investigated. Consensus could not be obtained by the experts on how the finding of an abnormal Allen’s test should be investigated, which is likely to be influenced by clinical contexts such as the age of the worker, duration of exposure, severity of blanching, and the local availability of specialised tests such as Doppler ultrasound and MR angiography. There was also a range of views as to whether a worker with primary RP should be allowed to work with vibrating tools. This was partly because of the lack of documented evidence that such an individual is at an increased risk of developing HAVS, and also because of the need to make adjustments in the light of a country’s disability discrimination legislation, such as an increase in the frequency of health surveillance and an individual’s ethical right of autonomy.

The purpose of health surveillance for HAVS is to educate workers about the harmful effects of vibration and how they should control their exposure to it. It is also used to assess the effectiveness of management controls, inform risk assessment and to prevent workers from reaching stage 3 vascular, or stage 2 or 3 neurological. Known occlusion of the radial, ulnar or palmar arteries may also need to be taken into consideration when advising a worker about work and trauma to the hands.

At all stages of HAVS, the worker’s ability to work safely should be considered. Advice regarding fitness and ongoing exposure should vary according to age, job requirements, workplace, tools used, ambient temperature, as well as the frequency and duration of symptoms, rather than by reaching a particular stage on the scale. Weakness of grip or loss of dexterity when the fingers are blanched or painful are difficult to measure but are as important as the occurrence of these effects when the fingers are not blanched; however, for obvious practical reasons they are rarely measured. For reasons of individual variability and subjectivity, the new scale takes no account of how workers may be disabled by their symptoms. Symptoms such as painful paraesthesia, cold intolerance, frequent or prolonged blanching and weakness of grip may be particularly troubling for the worker and should be taken into consideration when assessing fitness and safety to work.

## Conclusion

We recommend that frequency of blanching is dropped from the classification of HAVS and instead photographs of the fingers are taken during an attack of blanching for diagnostic confirmation of Raynaud’s phenomenon and for calculation of a blanching score. We recommend that two or more validated methods are used such as monofilaments, thermal aesthesiometry and vibrotactile thresholds to determine sensory perception loss in at least one finger. This new classification will have policy implications where the SWS is currently being used. There may be a need for specialist HAVS centres that can undertake clinical examinations and quantitative sensory tests with access to multi-segmental nerve conduction tests and vascular imaging.
